# Formulation and Optimization of Nanoemulsions Using the Natural Surfactant Saponin from *Quillaja* Bark

**DOI:** 10.3390/molecules25071538

**Published:** 2020-03-27

**Authors:** Tatiana B. Schreiner, Arantzazu Santamaria-Echart, Andreia Ribeiro, António M. Peres, Madalena M. Dias, Simão P. Pinho, Maria Filomena Barreiro

**Affiliations:** 1 Centro de Investigação de Montanha (CIMO), Instituto Politécnico de Bragança, Campus de Santa Apolónia, 5300-253 Bragança, Portugal; tatianas@ipb.pt (T.B.S.); asantamaria@ipb.pt (A.S.-E.); asribeiro@fe.up.pt (A.R.); peres@ipb.pt (A.M.P.); 2 Laboratory of Separation and Reaction Engineering – Laboratory of Catalysis and Materials (LSRE-LCM) Department of Chemical Engineering, Faculty of Engineering University of Porto, Rua Dr. Roberto Frias, S/N, 4200-465 Porto, Portugal; dias@fe.up.pt

**Keywords:** nanoemulsions, *Quillaja* bark saponin, high-pressure homogenization, design of experiments, zeta potential, particle size distribution

## Abstract

Replacing synthetic surfactants by natural alternatives when formulating nanoemulsions has gained attention as a sustainable approach. In this context, nanoemulsions based on sweet almond oil and stabilized by saponin from *Quillaja* bark with glycerol as cosurfactant were prepared by the high-pressure homogenization method. The effects of oil/water (O/W) ratio, total surfactant amount, and saponin/glycerol ratio on their stability were analyzed. The formation and stabilization of the oil-in-water nanoemulsions were analyzed through the evaluation of stability over time, pH, zeta potential, and particle size distribution analysis. Moreover, a design of experiments was performed to assess the most suitable composition based on particle size and stability parameters. The prepared nanoemulsions are, in general, highly stable over time, showing zeta potential values lower than −40 mV, a slight acid behavior due to the character of the components, and particle size (in volume) in the range of 1.1 to 4.3 µm. Response surface methodology revealed that formulations using an O/W ratio of 10/90 and 1.5 wt% surfactant resulted in lower particle sizes and zeta potential, presenting higher stability. The use of glycerol did not positively affect the formulations, which reinforces the suitability of preparing highly stable nanoemulsions based on natural surfactants such as saponins.

## 1. Introduction

Emulsions are formed by combining the right proportions of compounds with hydrophilic, lipophilic, and amphiphilic character. In other words, water, oil, and surfactant (or a combination of surfactants) are mixed to form a macroscopically homogeneous system from two or more immiscible compounds. In fact, because of its versatility [1,2], the technological application of emulsions is vast among industries that include food [3,4,5], pharmaceutics [6,7,8], and cosmetics [9,10,11]. Among the different types of emulsions, micro- and nanoemulsions present the most appealing properties due to their higher stability and possibility to serve as potential carriers of functionalities [12].

The surfactants are the components responsible for forming and stabilizing emulsion-based products. These molecules adsorb into oil–water interfaces during homogenization, reducing the interfacial tension and enhancing further droplet disruption. Additionally, the surfactants provide a protective layer around the droplets, which improves the long-term stability and inhibits their aggregation [13]. In this context, the use of surfactant mixtures, i.e., the addition of cosurfactants, is an interesting approach to obtain nanoemulsion systems at low surfactant concentration by reducing the interfacial tension and increasing the fluidity of the interface [14]. The most interesting cosurfactants consist of alcohols or glycols that have a low molecular weight and present a carbon chain between two and ten carbon atoms. Examples include glycerol, ethanol, propylene glycol, and n- butanol [15].

Nowadays, a significant challenge to be overcome in the field of emulsions is the introduction of natural products to act as surfactants [5,16,17]. This approach has been supported by the growth of consumer demands for more sustainable, natural, and environmentally friendly formulations, in line with the increasingly restrictive environmental legislation, in addition to the biocompatibility, biodegradability, and lower toxicity of such compounds. In this context, the research focused on products showing nature-friendly labels has been gaining attention. Particularly in the field of emulsions, this fact is related to the selection of a suitable surfactant, considering their high economic impact as well as the need of replacing the existing synthetic compounds by bio-derived low-cost alternatives [1,18].

Recent studies have been focused on the use of highly surface-active molecules like saponins [19], whose properties (biological and physicochemical) broaden their use to several applications. Saponin molecules comprise a hydrophilic region, containing rhamnose, galactose, xylose, fucose, or glucuronic acid, and a hydrophobic counterpart including gypsogenic or quillaic acid, whose combination ensures the amphipathic character of the molecule, enabling its surfactant behavior [20]. Moreover, the abundance of saponins in nature facilitates their commercial production [21,22] from a wide range of natural matrices, with *Quillaja* bark being one of the most used [23,24].

*Quillaja* bark saponins are natural surfactants obtained from the bark of *Quillaja saponaria* Molina trees. Their molecules are chemically built up of steroid aglycone moiety or triterpenoid attached by glycoside bonds into a sugar moiety [2,19,25]. This natural surfactant is allowed for human consumption as a food additive in several countries [26] and consequently has shown commercial applications in both food and cosmetic industries [24,27,28,29]. In recent years, saponins have gained interest in the preparation of nanoemulsions. The work of Ozturk et al. [30] identified that *Quillaja* saponin presented a high capacity for emulsifying and stabilizing nanoemulsions. A similar outcome was reported by Ralla et al. [19], utilizing the surfactant potential of saponins in nanoemulsions. More recently, Zhu et al. [31] performed a comparison between the *Quillaja* bark and the widely used synthetic surfactant Tween 80, where it was found that the saponin was highly surface-active and exhibited similar interfacial properties in comparison with the conventional synthetic Tween 80.

Regarding nanoemulsion production methods, the high-pressure homogenization (HPH) method has been given attention in the literature. This method is a readily available option at laboratory scale. It is suitable in terms of scale-up for industrial applications, turning HPH into an attractive strategy due to short processing times, avoiding the use of organic solvents, and its high efficiency in droplet size reduction [32,33]. Indeed, particle size and other parameters, including stability over time, zeta potential, viscosity, pH, and conductivity, are fundamental criteria for evaluating the quality and stability of the final emulsions [34].

In this context, the objective of this work is to analyze the formulation of nanoemulsions based on a natural surfactant (pure saponin from *Quillaja* bark) and the use of glycerol as cosurfactant, varying the percentage of the different components. Aiming to find the suitable formulation in terms of stability and particle size, a design of experiments (DOE) was conceptualized, varying the oil/water (O/W) ratio, total surfactant percentages, and saponin/glycerol ratio. The prepared emulsions were characterized, evaluating their stability over time, zeta potential, pH, and droplet size distribution. To the best of our knowledge, the use of a design of experiments to formulate stable nanoemulsions with saponin from *Quillaja* bark has never been applied before. In addition, the implementation of glycerol as a cosurfactant is a novel approach to be explored for obtaining highly stable nanoemulsions via simple, low-cost, and scalable methodologies.

## 2. Results and Discussion

### 2.1. Optimization of Emulsion Preparation Approach

**Figure 1:**
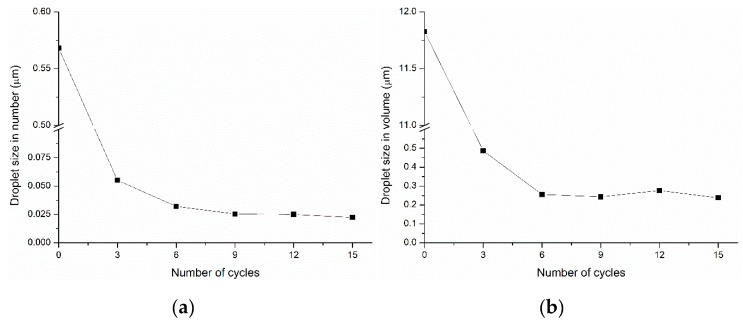
Influence of the number of cycles through high-pressure homogenization (HPH) on the particle size (**a**) in number and (**b**) in volume in a reference emulsion (O/W ratio of 20/80, 5 wt% saponin).

Oil-in-water nanoemulsions were prepared successfully by the HPH method. Regarding operational conditions, there is strong evidence that the droplet size approaches a constant value as the number of cycles increases [35]. Thus, a series of studies employing from 0 to 15 cycles were performed in order to find the optimum number of cycles in the emulsion preparation method before the preparation of samples. A reference composition consisting of an O/W ratio of 20/80 and 5 wt% saponin was employed for analyzing the particle size (in number and volume) of the emulsion considering the number of cycles, and the evolution is shown in Figure 1. It was observed that after six cycles, the droplet size in both number and volume became almost constant even when the number of cycles increased. Therefore, six cycles were employed for preparing the emulsions.

### 2.2. Characterization of Emulsions

**Figure 2:**
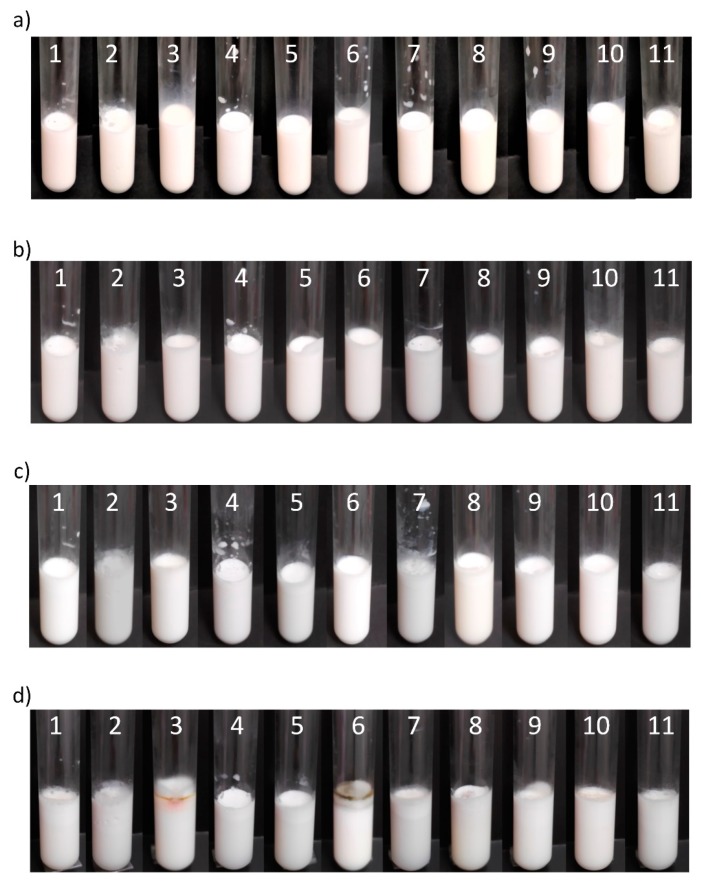
(**a**) Nanoemulsions prepared by HPH are shown on the preparation day. The evolution of their stability is shown after (**b**) 5, (**c**) 10 and (**d**) 30 days.

According to the proposed DOE, 11 different formulations were prepared, and their compositions are listed in Table 1. Furthermore, the emulsion appearances, as well as their evolution over time, are shown in Figure 2. The appearance of the emulsions on the day they were prepared can be observed in Figure 2a, where emulsions presented a milky appearance without showing phase separation.

As emulsion stability is an important parameter to be considered, the stability of the emulsions over time was evaluated. Figure 2b–d shows the emulsion appearances at 5, 10, and 30 days after preparation. It was observed that emulsions were generally stable for 30 days, with the exception of samples 2 and 11, which suffered a phase separation after 30 and 10 days, respectively. This phenomenon can be explained by two main reasons based on the sample composition: (i) since these two emulsions presented the highest amount of oil (O/W ratio of 30/70) and the lowest amount of surfactant (0.5 wt%), there was insufficient amount of surfactant to stabilize the amount of oil over time, thus causing the phase separation; (ii) regarding the cosurfactant content, sample 2 only contained saponin, while the glycerol in sample 11 represented half of the total surfactant. Considering that sample 2 resulted as more stable over time (30 days) than sample 11 (10 days), this indicates that the surfactant effect of pure saponin was more effective than when using glycerol as cosurfactant. Moreover, sample 7 also showed lower stability, presenting creaming formation after the 8th day due to the low amount of surfactant (0.5 wt%). Otherwise, considering emulsions with 30 days of preparation, microbial activity was observed in some samples, which can be caused by the absence of preservatives in the samples. After a longer time, all samples presented microbial presence.

**Figure 3:**
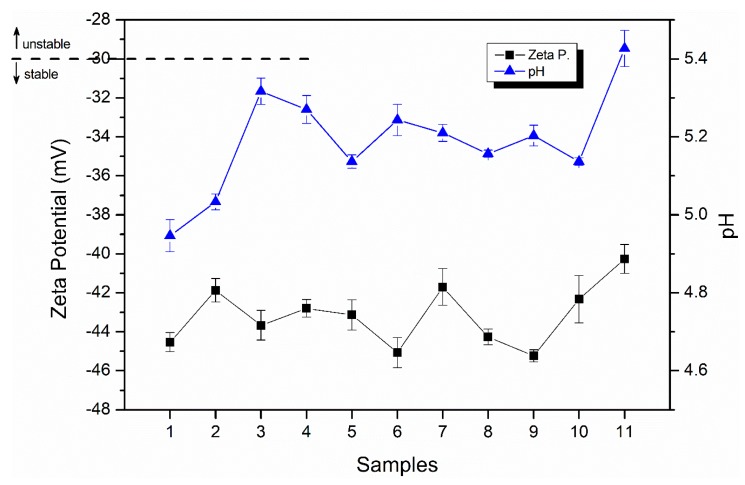
Zeta potential and pH values in the different samples.

The emulsions were also characterized in terms of pH and zeta potential, and the results are shown in Figure 3. Regarding pH measurements, an acidic character was observed in all samples. This fact was related to the properties of the constituents, both saponin (glucuronic, gypsogenic, and quillaic acids) and sweet almond oil (fatty acids), that hold low pH; therefore, it was not surprising to find values in the range of 4.95 to 5.43. The similarity in the pH values of the samples indicated that the composition did not significantly influence the pH.

Zeta potential is considered a useful parameter to predict the dispersion stability by measuring the surface charge of droplets [36]. This property can be defined as the value of electrokinetic potential associated to a realistic magnitude of surface charge [37]. It is known that to ensure the physical stability of nanoemulsions, the value of zeta potential should be far from zero, i.e., greater than 30 mV or less than −30 mV. The evaluated samples (Figure 3) had values ranging from −46 to −40 mV, with samples 2, 7, and 11 showing the highest values and the standard deviation being in the range of 0.3–1.2, comparable with values usually found in the literature [13,38]. These results indicated that the emulsions formed with saponin are highly stable, also corroborating the general stability over time previously shown in Figure 2. The negative surface potential value of emulsions stabilized by saponin was also observed in other works. This can be attributed to the carboxylic acid groups presented in the adsorbed saponin molecules [13,17]. Moreover, zeta potential results supported that saponin-coated particles were mainly stabilized by the electrostatic repulsion generated between the highly negatively charged droplets [31].

**Figure 4:**
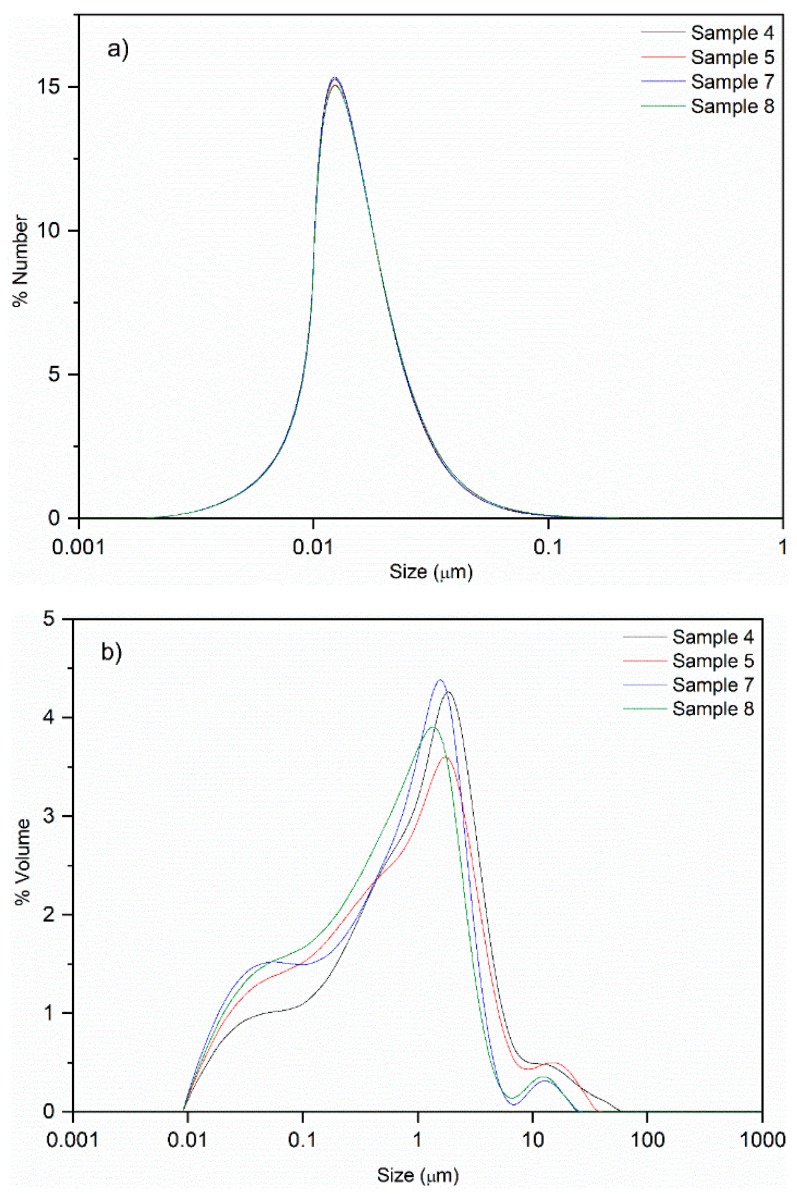
Particle size distribution of samples using an O/W ratio of 10/90 (4, 5, 7, and 8) in (**a**) number or (**b**) volume.

Another valuable property that has a direct effect on long-term stability, texture, and optical appearance of emulsions is the droplet size, with the average values, i.e., the particle size centered in 50% of the measured nanoparticles, obtained by dynamic light scattering (DLS; Section 3.5) being reported in Table 2. Particle size results in number showed very small particle sizes, ranging from 17 to 20 nm, while the data in volume varied between 1.1 and 4.3 µm, approximately. The large difference between the values in number and volume is based on the scattered light intensity in relation to the particle volume. When particle size and distribution are measured in volume, the larger particles give stronger intensity, even if they are present at small amounts [39], due to their surface volume comparing with smaller particles. Figure 4 shows both profiles (in number and volume) of the particle size distribution for samples 4, 5, 7 and 8 (all using O/W ratios of 10/90). The small peak close to 10 µm in the volume distribution curves (Figure 4b) indicated the presence of large particles that could cause the different outcomes in the number and volume profiles presented in practically all samples. It was also noticed that volume distribution profiles of the curves, according to the amount of oil, presented the same behavior (Figure 4b—10/90, Figure S1.1b—20/80, and Figure S1.2b—30/70). Complete information about size distribution for other samples and the corresponding D-values (D10, D50, and D90) for all samples (Table S1), are reported in the Supplementary Materials. However, the results in number presented quite similar values between the different samples, indicating that the small particles in all samples were alike, as can be seen in Figure 4a. For that reason, considering the decisive effect of the particle size in volume in the samples, this was chosen to perform the statistical analysis for stability studies (Section 2.3).

Analyzing the results shown in Table 2, some evidence was observed concerning the influence of the composition in the particle size. Firstly, when the same O/W ratio was used and the percentage of glycerol in the surfactant was the same, there was a significant decrease in the particle size as the amount of surfactant increased, suggesting the effectiveness of the surfactant in the emulsion formation and resulting in smaller droplets. For example, sample 4 (10/90 O/W, 50/50 saponin/glycerol, 0.5 wt% surfactant) and sample 5 (10/90 O/W, 50/50 saponin/glycerol, 1.5 wt% surfactant) presented 4.31 and 1.79 µm, respectively. Secondly, when the oil and surfactant contents were kept constant, the presence of glycerol increased the particle size of emulsion droplets, indicating the more effective surfactant effect of pure saponin, in comparison to glycerol. For instance, when comparing samples using O/W ratio of 10/90 and 0.5 wt% surfactant, namely sample 7 without glycerol (100% saponin) and sample 4 with 50/50 saponin/glycerol, the droplet size increased from 1.18 to 4.31 µm. The same behavior was observed for the O/W ratio of 30/70, while it was not feasible to conclude for 20/80, which is considered the central point of the design of experiments. This qualitative analysis is confirmed numerically in the next section.

### 2.3. Statistical Analysis

The factorial design is an essential tool for the identification of the experimental variables that play a significant role in particle size as well as in the zeta potential. Multiple linear regression models (MLRMs; Section 3.6) were performed to fit the response function (with Y corresponding to the particle size or zeta potential) using the experimental data collected. The developed models were established taking into account the general inference statistics for the global models as well as their coefficients at the usual significance levels (5%). However, it has to be kept in mind that [40,41]:

(i) Main effects are disregarded from the analysis if the hierarchy of the model is not affected.

(ii) Interaction effects are disregarded if they are not statistically significant and if their removal increases the overall significance of the model, even if it turns out into a nonhierarchical model (in this case, the final model equation in terms of actual factors is not provided since only hierarchical models are scale-independent and can be translated into actual units).

(iii) Effects are not removed if the final model is a ridge system where several local optimum points exist and the real stationary point is not inside the region of exploration for fitting the second-order model.

(iv) To ensure that the final model has a satisfactory prediction performance, the predicted coefficient of determination value must be higher than 0.25.

**Figure 5:**
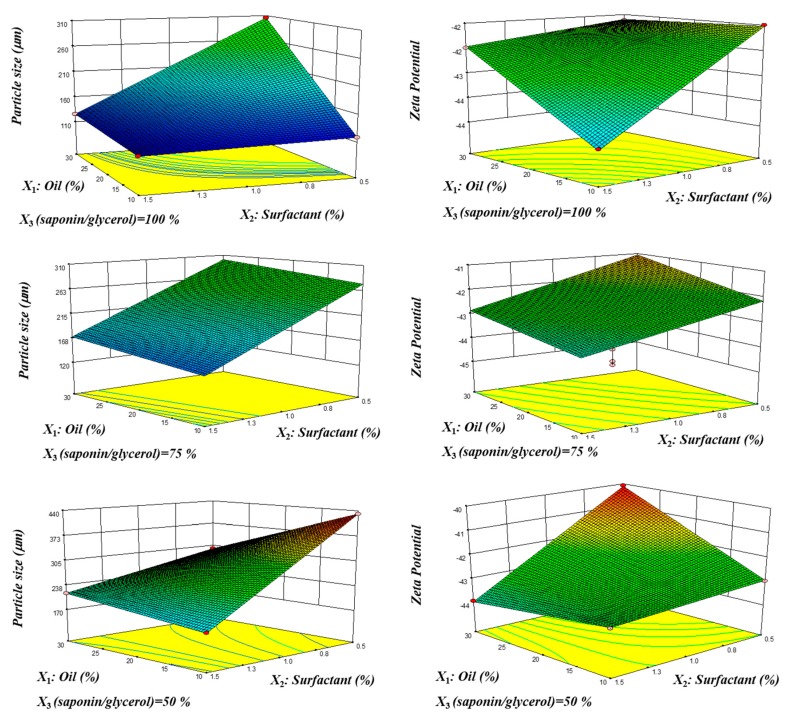
Response surface described by the model established for the particle size (in volume) and zeta potential in the experimental region evaluated: 3D plot and contour plots for oil and surfactant percentages at fixed saponin/glycerol percentages (minimum, central, and maximum). For representation purposes, the O/W ratio was considered as oil percentage, e.g., an O/W ratio of 10/90 is represented as 10% oil.

Bearing in mind the above-mentioned assumptions, two MLRMs were established to fit the response functions based only on the linear and interaction parameters originating reduced models. The statistical significance of the model and the coefficients of the response surface were evaluated using the analysis of variance (ANOVA) and Student’s *t*-test, respectively. Details of the models are given in Table 3 for the particle size and zeta potential, while the response surface described by the established model for each variable is shown in Figure 5.

Analyzing the DOE results, the significant influence of all selected compositions in the droplet size of the emulsions can be noted, with the amount of surfactant being the most relevant parameter. The reduction of particle size was also observed with the decrease of the percentage of oil and/or the increase of the amount of surfactant. The incorporation of glycerol as cosurfactant had a negative impact on particle size reduction, as previously discussed in Section 2.2.

Zeta potential was the other selected property in order to evaluate the nanoemulsions stability behavior. The global change of that variable within all performed experiments was small, and, possibly due to that fact, only a few parameters showed a significant effect, including oil and surfactant percentages, which showed opposite effects. While the increase of the surfactant percentage decreased zeta potential values, the opposite tendency was observed in the case of oil content. Even if some of the regression parameters did not present statistical significance at a 5% significance level, they were kept in the model to ensure the goodness of the fit. Nevertheless, as expected, the prediction performance was not as accurate as in the particle size prediction.

In Table 3, it can be observed that the curvature was significant for both particle size and zeta potential parameters. This fact indicated that the chances of this occurring due to noise (pointing out the possible need to enlarge the experimental design in order to include higher-order coefficients into the model that could account for this deviation to linearity) are only 0.07% and 0.13% for particle size and zeta potential, respectively. Regarding the lack of fit, it was not significant relative to the pure error, meaning that the proposed model accurately fits the experimental data and that the chances of this occurring due to noise were 92.93% and 84.25% for droplet size and zeta potential, respectively. Furthermore, considering the quality parameters, namely adequate precision, which is a measure of the signal-to-noise ratio (with a ratio greater than 4 being desirable), values of 41.83 and 18.03 were obtained in both cases, indicating that the model could be used to navigate the design space. Moreover, the magnitude of R², R²_adj_, and R²_pred_ suggested a very satisfactory predictive capability. Comparing these main parameters discussed above, it can be stated that all parameters studied showed slightly better precision for the particle size assessment, a fact that could be related to the small variance in the zeta potential of measured samples.

## 3. Materials and Methods

### 3.1. Materials

The natural surfactant, a pure saponin from *Quillaja* bark (99.9%), was purchased from AppliChem GmbHn (Darmstadt, Germany), with pH between 4.5 and 5.5 and sapogenin content in the range of 10%–14%. Cosurfactant glycerol (pharmaceutical grade) was purchased from LabChem (LaborSpirit, Lisbon, Portugal).

For the oil phase, a cosmetic-grade sweet almond oil (JMGS, Odivelas, Portugal) was used. It had a density of 0.913 g/cm^3^ (at 25 °C) and a saponification value of 192 mg KOH/g. It presented palmitic acid (4.9%), palmitoleic acid (0.1%), stearic acid (2.8%), oleic acid (65.3%), linoleic acid (25.2%), and linolenic acid (0.1%) in its composition.

Deionized water was also used as the aqueous phase for all experiments.

### 3.2. Emulsion Preparation and Stability

Oil-in-water emulsions of different compositions were prepared to achieve O/W ratios of 10/90, 20/80, and 30/70 (*w*/*w*). The total surfactant amounts used were 0.5, 1.0, and 1.5 wt%, referring to the total oil plus water weight, whereas the variation of the cosurfactant (glycerol) was between 0 and 50 wt% (surfactant-basis). For the study, 40 mL of each sample were prepared. The importance of considering the addition sequence of the components in the nanoemulsion preparation method should be noted. Briefly, the surfactant was first added to the oil phase and homogenized. Then, this oil phase and water were mixed in a flask and blended using an Ultraturrax for 3 min at 11,000 rpm speed, and a coarse emulsion was formed. Afterwards, the coarse emulsion was subjected to a high-pressure homogenization (HPH) (Avestin Emulsiflex C3) protocol of six cycles at a homogenization pressure of 100 MPa. During the process, there was a tendency to increase the temperature of the sample, so an attached heat exchanger was used to keep the temperature constant.

The prepared samples were stored in the dark at room temperature (20 °C), and the storage stability was analyzed after 1, 5, 10, and 30 days. The samples were stored in the dark at room temperature (20 °C).

### 3.3. pH Measurements

Characterization of emulsions was carried out by pH measurements using PH-metro InoLab 720 WTW. All tests were performed directly in the samples at 25 °C, in triplicate, and mean values were used.

### 3.4. Zeta Potential

Stability of emulsions was analyzed by determining particle surface charge characteristics using an electrophoresis instrument (Zetasizer Nano-ZS90, Malvern Instruments, Worcestershire, UK). Samples were diluted (between 1:25 and 1:40) with deionized water before the analysis to avoid multiple scattering effects and were analyzed in triplicate.

### 3.5. Particle Size Determination

Emulsion particle size and distribution were obtained using dynamic light scattering (DLS) equipment (Mastersizer 2000, Malvern Instruments, Worcestershire, UK). The refractive indexes used for the dispersed (oil) and continuous phases (water) were 1.47 and 1.33, respectively. Particle size and distribution percentages in volume and number were determined at room temperature by averaging five measurements for each sample.

### 3.6. Statistical Analysis

The optimal oil and surfactant percentages, as well as the saponin/glycerol ratio, were the variables selected to evaluate the best operating conditions to minimize the particle size (in volume) and obtain zeta potential values in the stable range. The analysis was carried out using a 2^k^ full-factorial design with three factors and three replicates of the central point. The three independent factors (oil, surfactant, and saponin/glycerol levels, corresponding to the actual factors *x*_1_, *x*_2_, and *x*_3_, respectively) were studied at three levels (−1, 0 and +1), as shown in Table 4, resulting in 11 experiments that were carried out randomly.

For the statistical treatment, the actual factors were coded according to the following equation:

(1) $$X_{i}=\frac{x_{i}-x_{0}}{\Delta x_{i}},\;\mathrm{with}\;i=1,2,3$$

where *X_i_* is the coded value of the independent factor, *x_i_* is the real value of the independent factor, *x*_0_ is the real value of the independent factor at the central point, and Δ*x_i_* is the step change value.

It was expected that a first-order linear equation could explain the behavior of the system and second-order coefficients (with the latter being related to the interactions); this equation was used for predicting the optimal particle size or zeta potential (*Y*_1_ and *Y*_2_, respectively), based on the coded values of the independent factors (*X_i_*):

(2) $$Y_{k}=\beta_{0,k}+\overset{3}{\underset{i=1}{\sum }}\left(\beta_{i,k}X_{i}\right)+\overset{3}{\underset{i<j}{\sum }}\left(\beta_{ij,k}X_{i}X_{j}\right)+\beta_{123,k}X_{1}X_{2}X_{3},\;\mathrm{with}\;k=1,2$$

where *Y* is the predicted response; the β coefficients are the first-, second- and third-order parameters whose values are to be determined using multiple linear regression models (MLRMs), and the statistically significant ones are selected using a stepwise method. The first order parameters are related to the screening process, the second-order ones are related with the model curvature, and the third-order parameters are due to asymmetry issues.

Design-Expert 6.0.6., trial version, was used for the experimental design and regression analysis of the experimental data. The significance of the regression model was evaluated using analysis of variance (ANOVA). The quality of the fit obtained using the regression model equation was statistically checked using two diagnostic residuals: the multiple or adjusted coefficients of determination (R^2^ or R^2^_adj_, respectively) and the predicted coefficient of determination (R^2^_pred_). The R^2^ and R^2^_adj_ values describe the goodness of fit, giving an idea of how well current runs can be reproduced by the mathematical model. The R^2^_pred_ value describes the goodness of prediction, showing how well new experiments can be predicted using the mathematical model. R^2^ or R^2^_adj_ greater than 0.75 and R^2^_pred_ values higher than 0.60 usually indicate that the model is good, and R^2^_pred_ values lower than 0.25 indicate that the model is useless [15]. The discrimination ability of the model was also inferred by calculating the adequate precision value, which compares the range of the predicted values at the design points to the average prediction error. A value greater than 4 is envisaged to assure adequate model discrimination. The significance of the regression coefficients was tested using a *t*-test. Finally, the contour plots obtained from the fitted quadratic or cubic model were also used to infer the optimal experimental conditions keeping the independent factors within the experimental range studied.

## 4. Conclusions

In this work, the formulation of emulsions using saponin as surfactant and glycerol as cosurfactant was carried out. Different compositions were prepared by varying the O/W ratio, the total surfactant content, and the surfactant composition (expressed as saponin/glycerol ratio). The prepared samples were analyzed in terms of stability, pH, zeta potential, and particle size and distribution in number and volume. Analysis of stability over time showed stable emulsions after 30 days, except for samples with a low amount of surfactant (0.5 wt%). Results of pH measurements proved the acidic character of samples, and zeta potential assays indicated negative values (lower than −40 mV) corroborating the high stability of the samples. Average particle size in number showed low values (around 20 nm) with a similar particle size distribution behavior, which was mainly influenced by the O/W ratio. Results in volume indicated larger values in the micro-scale range, pointing out the heterogeneity of the sample in terms of size distribution.

To complement the study, a design of experiments was performed in order to analyze the effect on particle size (in volume) and zeta potential. Concerning these parameters, the most suitable formulation for sweet almond oil based emulsions stabilized with saponin was the one containing a low O/W ratio, namely 10/90, combined with an amount of surfactant around at least 1.5 wt%. Moreover, it should be noted that the use of glycerol as cosurfactant did not positively affect the quality of the nanoemulsions in this work, which was not an expected behavior since it had been reported in other works that the combination of *Quillaja* saponin with cosurfactants offers the chance to improve the emulsion stability with a lower amount of surfactant [1]. Thus, more studies focusing on the interactions of *Quillaja* saponin with cosurfactants are still needed. However, this outcome concerning the glycerol also highlighted the effectiveness of saponin itself acting as a surfactant, leading to emulsions with lower particle size.

The results of this work pointed out the need for further studies concerning the formulation of nanoemulsions from natural surfactants. Further studies are still required to establish the basis of novel formulations, in which it is essential to systematically characterize and compare the ability to form and stabilize emulsions, besides additional analysis, such as microbiological stability and lipid oxidation.

## Figures and Tables

**Table 1:** Sample compositions used in the design of experiments (DOE).

	1	2	3	4	5	6	7	8	9	10	11
O/W (*w*/*w*)	20/80	30/70	30/70	10/90	10/90	20/80	10/90	10/90	20/80	30/70	30/70
Surfactant (wt%)	1.0	0.5	1.5	0.5	1.5	1.0	0.5	1.5	1.0	1.5	0.5
Saponin/Glycerol (*w*/*w*)	75/25	100	50/50	50/50	50/50	75/25	100	100	75/25	100	50/50

**Table 2:** Particle size averages determined by dynamic light scattering (DLS) in number and volume.

	1	2	3	4	5	6	7	8	9	10	11
Size number (nm)	20.67	19.03	17.97	18.06	18.29	19.17	17.98	18.36	18.31	18.50	17.74
Size volume (µm)	2.09	3.05	2.15	4.31	1.79	1.47	1.18	1.12	1.25	1.25	2.97

**Table 3:** Regression parameters (β coefficients) of the optimal multiple linear regression models (MLRMs) established for the particle size in volume (µm) and zeta potential (mV) using a stepwise variable selection method for the 2^3^ experimental design and respective model quality parameters.

Source	Particle Size in Volume (µm)		Zeta Potential (mV)	
	β Coefficient (Coded Factors)	*p*-Value	β Coefficient (Coded Factors)	*p*-Value
**Model**	----	0.0006	----	0.0161
Intercept	+222.90	----	−42.50	----
*X*_1_—oil (%)	+12.71	0.0280	+0.47	0.0217
*X*_2_—surfactant (%)	−65.09	0.0003	−0.85	0.0042
*X*_3_—saponin/glycerol (%)	−57.72	0.0004	−0.04	0.7489^1^
*X*_1_ *X*_3_	+37.42	0.0013	−0.12	0.3405^1^
*X*_2_ *X*_3_	+18.44	0.0102	+0.09	0.4730^1^
*X*_1_ *X*_2_ *X*_3_	−43.01	0.0009	+0.65	0.0091
Curvature	-----	0.0007	-----	0.0013
Lack of fit	----	0.9293	----	0.8425
**Quality parameter**	**Value**		**Value**	
Adequate Precision	41.833		18.030	
R^2^	0.9973		0.9757	
R^2^_adj_	0.9920		0.9272	
R^2^_pred_	0.9932		0.9082	

^1^ Parameter not statistically significant at a 5% significance level kept in the model to ensure hierarchy and the good of fitness.

**Table 4:** Parameter definition for design of experiments.

Variable	Symbol	Coded (*X*_i_) Variable Level		
		−1	0	+1
Oil (%)	*x*_1_	10	20	30
Surfactant (%)	*x*_2_	0.5	1.0	1.5
Saponin/glycerol (%)	*x*_3_	50	75	100

## Supplementary Materials

The following are available online, Figure S1.1. Particle size distribution of samples using a O/W of 20/80 (1, 6 and 9) in (a) number or (b) volume., Figure S1.2. Particle size distribution of samples using a O/W of 30/70 (2, 3, 10 and 11) in (a) number or (b) volume.
