# Supplementary material for: Formulation and Optimization of Nanoemulsions Using the Natural Surfactant Saponin from Quillaja Bark
Source: Molecules. 2020 Mar 27;25(7):1538. doi: 10.3390/molecules25071538 (PMC7181021; doi:10.3390/molecules25071538)
Supplement: Supplementary file 1 [file molecules-25-01538-s001.pdf]

Supplementary Material for

Formulation and Optimization of Nanoemulsions using the Natural Surfactant *Saponin* from *Quillaja* Bark

Tatiana B. Schreiner [1,2], Arantzazu Santamaria-Echart [1], Andreia Ribeiro[1,2], António M. Peres [1], Madalena M. Dias [2], Simão P. Pinho [1,*], Maria F. Barreiro [1, †]

[1] Centro de Investigação de Montanha (CIMO), Instituto Politécnico de Bragança, Campus de Santa Apolónia, 5300-253 Bragança, Portugal;

[2] Laboratory of Separation and Reaction Engineering – Laboratory of Catalysis and Materials (LSRE/LCM) Department of Chemical Engineering, Faculty of Engineering University of Porto, Rua Dr. Roberto Frias, S/N, 4200-465 Porto, Portugal; e-mail@e-mail.com

[*] **Corresponding author:** e-mail: spinho@ipb.pt.
[†] **Corresponding author:** e-mail: barreiro@ipb.pt.

S1. Particle size distribution in number and volume obtained by DLS.

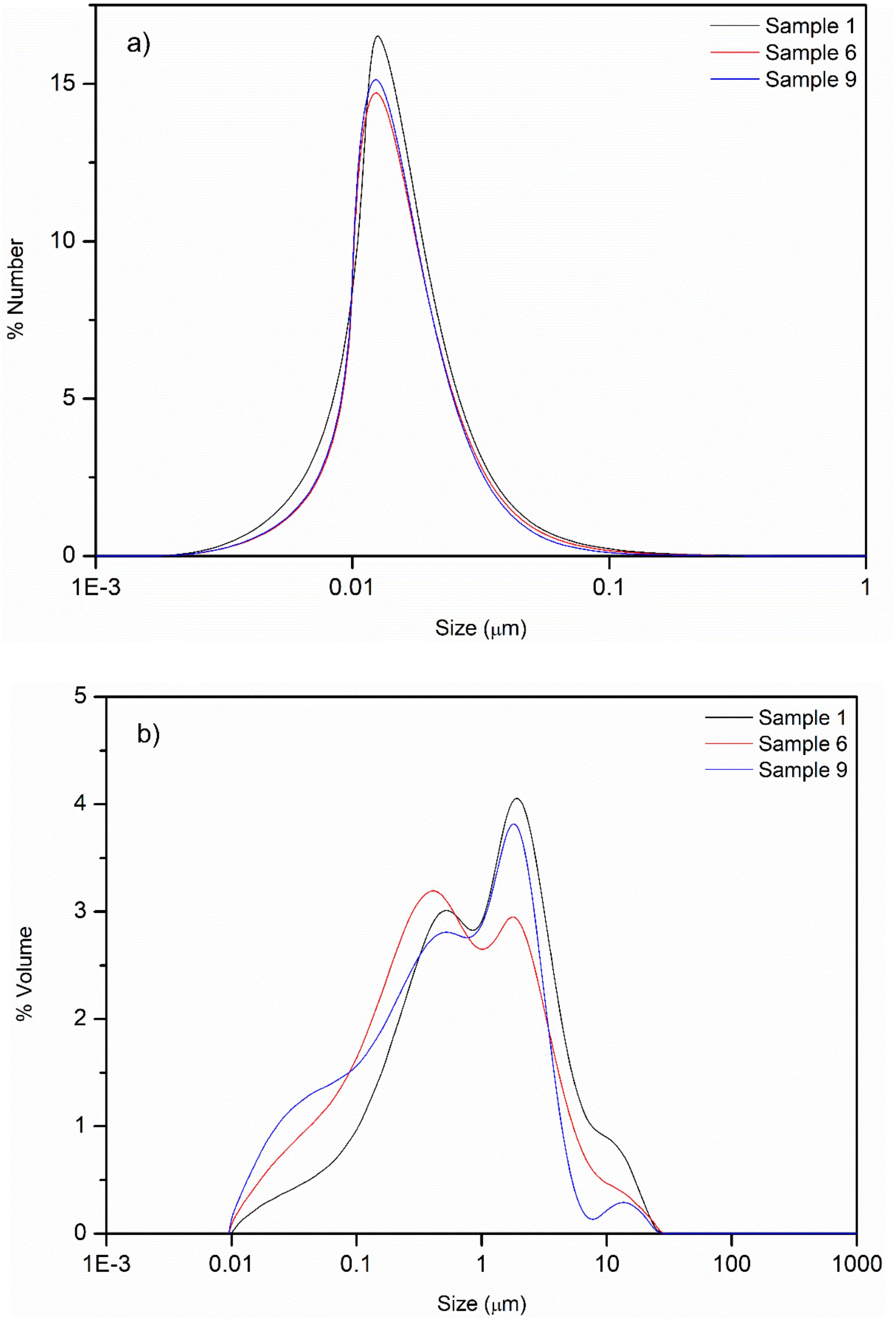

Figure S1.1. Particle size distribution of samples using a O/W of 20/80 (1, 6 and 9) in (**a**) number or (**b**) volume.

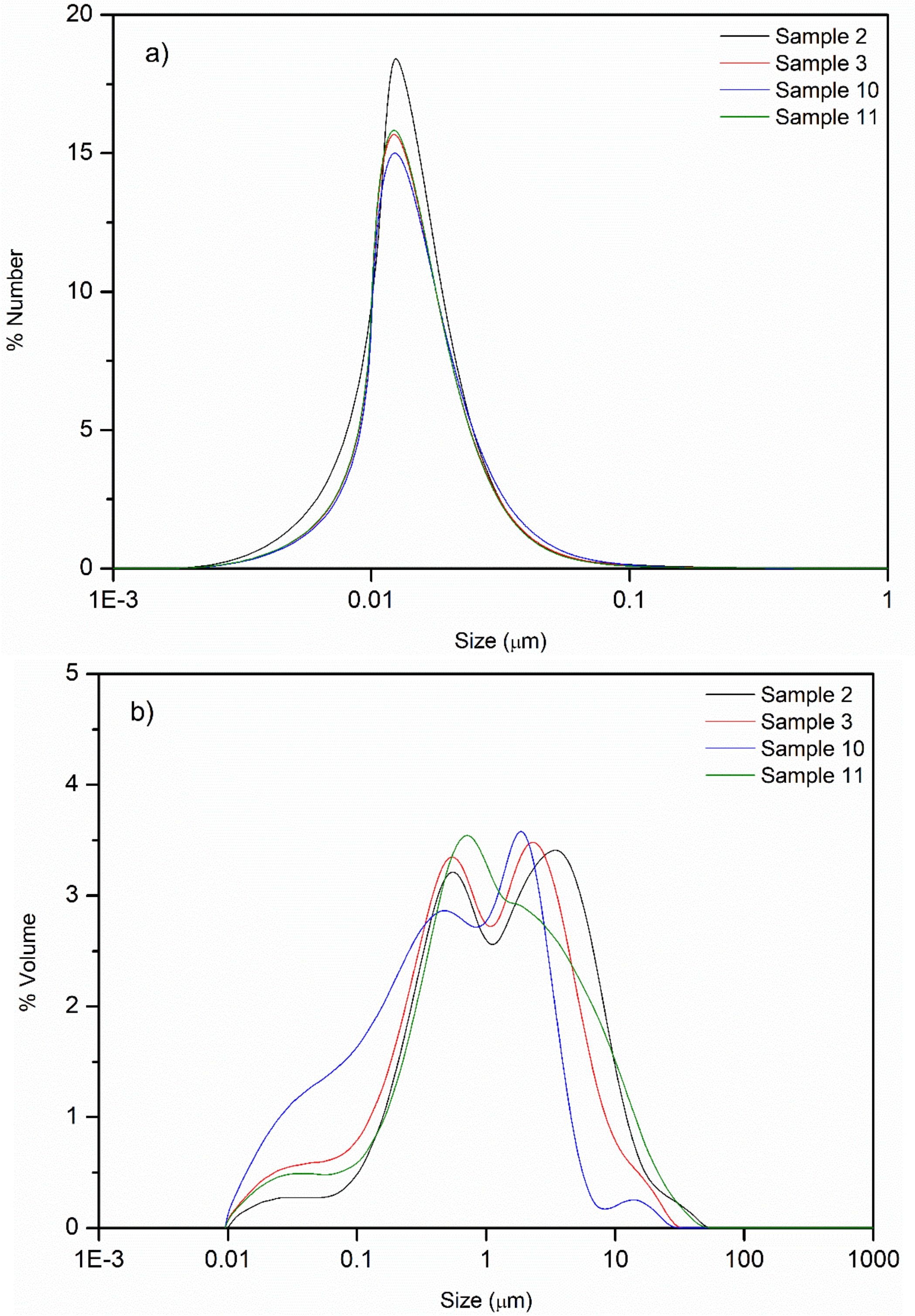

Figure S1.2. Particle size distribution of samples using a O/W of 30/70 (2, 3, 10 and 11) in (**a**) number or (**b**) volume.

Table S1. Determined D-Values (D10, D50 & D90) in number and volume.

Sample	D10	D50	D90
		Size in Number (nm)	
1	12.3 ± 0.003	17.0 ± 0.023	34.4 ± 0.206
2	12.2 ± 0.002	16.2 ± 0.009	29.5 ± 0.022
3	11.1 ±0.001	15.6 ± 0.003	29.3 ± 0.029
4	11.2 ± 0.001	15.8 ± 0.004	30.0 ± 0.021
5	11.2 ± 0.001	15.9 ± 0.004	30.7 ± 0.029
6	11.2 ± 0.001	16.1 ± 0.004	32.4 ± 0.036
7	11.2 ± 0.000	15.8 ± 0.001	30.0 ± 0.004
8	11.2 ± 0.001	15.9 ± 0.004	30.8 ± 0.026
9	11.2 ± 0.001	15.9 ± 0.003	30.6 ± 0.025
10	11.2 ± 0.001	15.9 ± 0.004	31.0 ± 0.025
11	11.1 ± 0.000	15.5 ± 0.002	28.7 ± 0.008
		Size in Volume (μm)	
1	0.14 ± 0.003	1.09 ± 0.006	5.28 ± 0.053
2	0.23 ± 0.003	1.53 ± 0.004	7.97 ± 0.028
3	0.12 ± 0.002	1.02 ± 0.004	5.61 ± 0.033
4	0.06 ± 0.000	1.02 ± 0.004	4.40 ± 0.035
5	0.05 ± 0.001	0.73 ± 0.005	3.97 ± 0.026
6	0.07 ± 0.000	0.57 ± 0.001	3.76 ± 0.003
7	0.04 ± 0.000	0.68 ± 0.002	2.67 ± 0.005
8	0.04 ± 0.000	0.57 ± 0.002	2.48 ± 0.006
9	0.05 ± 0.000	0.60 ± 0.003	2.99 ± 0.010
10	0.05 ± 0.000	0.58 ± 0.002	3.08 ± 0.010
11	0.16 ± 0.002	1.20 ± 0.003	8.55 ± 0.043
